# Data on the impact of physical exercise treatment on depression and anxiety in a psychiatric hospital for adolescents

**DOI:** 10.1016/j.dib.2022.108165

**Published:** 2022-04-12

**Authors:** Arnaud Philippot, Vincent Dubois, Kate Lambrechts, Denis Grogna, Annie Robert, Ugo Jonckheer, Wagdan Chakib, Alexandre Beine, Yannick Bleyenheuft, Anne G De Volder

**Affiliations:** a Université catholique de Louvain, Institute of Neuroscience, MSL-In Laboratory, Brussels, Belgium; b Psychiatric Hospital Area+/Epsylon ASBL, Brussels, Belgium; c Cliniques Universitaires Saint-Luc, Pediatric Neurology Service, Brussels, Belgium; d Université catholique de Louvain, Institut de recherche expérimentale et clinique, EPID Epidemiology and Biostatistics Research Pole; e Environmental, Aging (Integrative) Physiology Laboratory, Haute Ecole Bruxelles-Brabant (HE2B), Brussels, Belgium

**Keywords:** Exercise, Depression, Anxiety, Psychiatry, Adolescence, Adolescent medicine

## Abstract

The present data article provides a dataset of psychological scores, additional description of used measures, and descriptive data of participants related to the research article entitled “Impact of physical exercise on depression and anxiety in adolescent inpatients: a randomized controlled trial” (Philippot et al., 2022). This randomized controlled trial aimed at assessing the effect of add-on treatment with structured physical exercise compared to social relaxation activities in a clinical population of adolescents hospitalized for depression and anxiety in a psychiatric hospital. A group of 40 adolescents was randomly assigned to either a physical exercise or a control program three to four times per week over six weeks. The primary outcome was the Hospital Anxiety Depression Scale (HADS) for evaluation of depression and anxiety symptoms. Secondary outcomes were psychological self-assessments (The Zung Self-Assessment Depression Scale (SDS), Beck's abbreviated Depression Inventory (BDI-13), The Child Depression Inventory (CDI), The State-Trait Anxiety Inventory (STAI)), diagnostic interview (Hamilton Depression Rating Scale), and physical examinations (an adapted version of the Astrand-Rhyming Sub-Maximal Effort Test and BMI measures). These questionnaires and tests were filled at baseline and after intervention.

## Specifications Table

**Table:** 

Subject	Health and Medical Sciences
Specific subject area	Exercise treatment for clinical depression and anxiety in a psychiatric hospital for adolescents
Type of data	Table Dataset
How data were acquired	**Dataset:** Data were collected by self-reports completed by the participants, by semi-structured interviews with a psychiatrist, and by physical tests on a cycloergometer with anthropometric measurements. **Table:** Characteristics were acquired directly from servers with electronic medical records
Data format	Raw Analyzed
Description of data collection	*Self-reported questionnaires (HADS, SDS, BDI, CDI, STAI) were* completed by the participant in a quiet room in presence of the experimenter who gave instructions to ensure that conditions were the same for all participants. *HAM-D questionnaires were* completed by a psychiatrist during a semi-structured interview with the participant in a quiet room. *For physical condition assessment,* VO2Max was evaluated by cycle ergometer with an adapted version of the Astrand-Rhyming Sub-Maximal Effort Test, BMI was calculated as weight in kilograms divided by the square of height in meters. Characteristics of participants are providing additional information on origins, education, and family status.
Data source location	Institution: AREA+, Epsylon ASBL City/Town/Region: Brussels Country: Belgium Latitude and longitude (and GPS coordinates, if possible) for collected samples/data: Q8VW+39 Uccle
Data accessibility	Data are provided with the present article and available in the Psycharchives repository on this persistent identifier; https://doi.org/10.23668/psycharchives.5625.
Related research article	Arnaud Philippot, Vincent Dubois, Kate Lambrechts, Denis Grogna, Annie Robert, Ugo Jonckheer, Wagdan Chakib, Alexandre Beine, Yannick Bleyenheuft, Anne G De Volder, (2022) Impact of physical exercise on depression and anxiety in adolescent inpatients: a randomized controlled trial. *Journal of Affective Disorder.*

## Value of the Data

• The database describes the effect of exercise add-on treatment on clinical symptoms of depression, anxiety, and physical condition in inpatients in a psychiatric hospital for adolescents, using a randomized controlled trial (RCT) design that is the best procedure for inference.
• The data can provide better knowledge for researchers on the efficiency of exercise treatment or to observe the efficiency of hospitalization for depression and anxiety in adolescents or more globally gather information about the severity of symptoms in inpatients in a psychiatric setting.
• The data can provide reference on the impact of exercise treatment on psychological symptoms and cardiovascular conditions to develop future research study design in the field of exercise in mental health.
• All psychological data could be compared with physical data that correspond to the cardio-vascular condition.

## 1 Data Description

From an RCT evaluating a physical exercise intervention compared to a control group, this Data in Brief provides psychological and physical scores at baseline and after intervention for inpatients in AREA+, a psychiatric hospital for adolescents. Participants completed self-reports for depression (HADS D, CDI, BDI, SDS) and anxiety symptoms (HADS A and STAI). A semi-structured interview was conducted by a psychiatrist to provide a diagnosis of depression (HAM-D). Physical condition was assessed using BMI and VO2max estimated from an adapted version of the Astrand-Rhyming Sub-Maximal Effort Test. In each group, two participants did not perform the test for estimating the VO2Max because of technical problems or a malaise on the day of physical testing.

Additional descriptive characteristics of participants are provided on age, gender, origin, education, and family history (Table 1).

**Table 1:** Additional characteristics of participants modified from Philippot et al. [1].

	Total *n* = 40		Control *n* = 20		Physical exercise *n* = 20	
**Age**	15.4 ± 1.6 (SD)		15.2 ± 1.5 (SD)		15.5 ± 1.8 (SD)	
**Gender**						
Male	15	38%	7	35%	8	40%
Female	25	63%	13	65%	12	60%
**Origin**						
European	27	68%	11	55%	16	80%
European/Middle-Eastern	7	18%	4	20%	3	15%
European/African/South American	6	15%	5	25%	1	5%
**Education**						
School-level						
≤ 1st cycle	15	38%	8	40%	7	35%
≥ 2nd cycle	23	58%	11	55%	12	60%
Unknown	2	5%	1	5%	1	5%
School Attendance						
Regular	8	20%	4	20%	4	20%
Altered	8	20%	3	15%	5	25%
Drop out>3 months	11	28%	7	35%	4	20%
Drop out>6 months	13	33%	6	30%	7	35%
≥ 1 failed year	26	65%	14	70%	12	60%
Experience of bullying	22	55%	11	55%	11	55%
**Family**						
Status						
Parents living together	11	28%	5	25%	6	30%
Custody by 50% Father /50% Mother	3	8%	2	10%	1	5%
Custody by mainly/only one parent	23	58%	11	55%	12	60%
Other*	3	8%	1	5%	2	10%
History						
Mental health issues**	19	48%	10	50%	9	45%
Alcohol/Substance abuse	10	25%	4	20%	6	30%
Disabled physical disease***	8	20%	4	20%	4	20%
Parent Death****	14	35%	7	35%	7	35%
Youth service support	22	55%	11	55%	11	55%
Preferential tariff or precariousness	13	33%	5	25%	8	40%

* Living in youth center (legal decision)

** Depression/schizophrenia/bipolar trouble in family

*** Cancer, stroke, neurological disease in family

**** Death by accident, severe disease or suicide, concerning closed family

Modified from the co-submission paper: A. Philippot, V. Dubois, K. Lambrechts, D. Grogna, A. Robert, U. Jonckheer, W. Chakib, A. Beine, Y. Bleyenheuft, A.G. De Volder, Impact of physical exercise on depression and anxiety in adolescent inpatients: A randomized controlled trial, Journal of affective disorders (2022).

## 2 Experimental Design, Materials and Methods

### 2.1 Design

The design used in the dataset was a randomized controlled trial aimed at evaluating the add-on effect of structured and supervised physical exercise compared to a social relaxation activity in a psychiatric hospital for adolescents for the treatment of clinical depression and anxiety. The participants completed the psychological and physical tests at the baseline three days before the beginning of the programs. We used a fixed randomization procedure. A sequential list of 40 numbers was set up and sealed before beginning the trial, using random permuted blocks of size 2 or 4 at random. Pair or odd numbers in the list corresponded to each of the two treatment arms. Each time an adolescent was recruited, the principal investigator was contacted by email with an entry badge number, blind to identity as well as to data of participant, and he responded by email either pair or odd, according to his list. The two interventions entailed four sessions of 1 hour per week during five to six weeks to reach a total of twenty hours of activity per program. Participants completed on average 18.2 (SD 2.2) sessions in the control group and 18.4 (SD 2.1) sessions in the exercise group. The mean attendance rate was 76% for the exercise group and 79% for the control group. Adolescents completed the same psychological and physical measures to evaluate their evolution three days after interventions.

### 2.2 Setting

Data were collected in AREA+, Epsylon ASBL, a psychiatric hospital offering care for adolescents.

The siteweb is: https://www.epsylon.be/index.php/hospitalisations/area.

### 2.3 Primary outcomes

The Hospital Anxiety & Depression Scale (HADS) is a reliable and valid questionnaire [2] for hospitalized populations, consisting of seven items (rated 0, 1, 2 and 3) related to anxiety (subscale A) and seven others to depression (subscale D), thus providing two scores. The internal reliability of HADS was good (Cronbach's alpha = 0.79 to 0.86). In each subscale, the score ranges between 0 and 21, with a diagnostic interpretation as no symptoms (0–7), probably pathological (8–10), considered clinical (11–21). From 57 studies including 10 664 participants and 1048 with major depression, a recent systematic review demonstrated a sensitivity (0.74, 95% Confiance Interval (CI): 0.68 to 0.79) and specificity (0.84, 95% CI: 0.81 to 0.87) for a cut-off value of eight or higher [3]. Results from the French version of HADS showed good consistency and good psychometric properties in Western cultures [4] and in the primary care population in Quebec [5], according to other studies in different countries [4,5].

### 2.4 Secondary outcomes

The Hamilton Depression Rating Scale (HAM-D) is a semi-structured diagnostic interview evaluating depression [6] using the criteria of the diagnostics and statistics manual (DSM) [7], that was administered by psychiatrists who were blind to the therapy group in this study. The HAM-D is still considered as a gold standard for this purpose because it is multidimensional, easy to use by psychiatrists, and has adequate internal and inter-rater reliability [8]. The score ranges from 0 to a maximum of 52, and the interpretation is: no depression (0–7), mild depression (8–16), moderate depression (17–23) or severe depression (24–52) [9].

The Child Depression Inventory (CDI) is a 27-item self-assessment tool used in longitudinal follow-up at school that provides a score based on the participant's statements over the preceding two weeks [10]. From 54 studies (66 data points, 34,542 participants), the CDI shows good reliability (0.89, CI: 0.86–0.92), sensitivity (0.80, 95% CI: 0.76–0.84), specificity (0.78, 95% CI: 0.74–0.83), and it has a low level of bias [11]. The score ranges from 0 to a maximum of 54, and its interpretation is: normal, i.e. mean score in the general population (0–9), moderate (10–18), or pathological (19-54).

Beck's abbreviated Depression Inventory (BDI-13) [12] includes 13 multiple-choice questions. The BDI-13 has a high internal consistency (0.86 for a non-clinical population) and demonstrates good internal reliability (0.89, 95% CI: 0.86-0.92), sensitivity (0.80, 95% CI: 0.76-0.84), and specificity (0.78, 95% CI: 0.74–0.83) [11]. The score ranges from 0 to a maximum of 39, with an interpretation of non clinical score if below cut-off (< 8) or pathological score if above cut-off (≥ 8).

The Zung Self-Assessment Depression Scale (SDS) [13] allows to assess the severity of depression according to 20 items. The internal consistency is adequate (Cronbach's alpha = 0.79). A meta-analysis demonstrated that the symptom-specific depressive factors in SDS seem to be relatively robust and well established [14].The score ranges from 0.25 to a maximum of 1, and its interpretation is: no depression (0.25–0.49), mild depression (0.50–0.59), moderate depression (0.60–0.69), severe depression (0.70-1).

The State-Trait Anxiety Inventory (STAI) [15] consists of two subscales with twenty items each, consisting of the STAI, form Y-A, which assesses the recent state (i.e. how participants felt during the test week) and the STAI, form Y-B, which assesses the long-term trait anxiety (i.e., how participants felt in the past year). This test has a high reliability (Cronbach's alpha = 0.86). The internal consistency and test-retest reliability (two weeks) were good among a western Spanish sample in a non-clinical and clinical sample [16]. The score ranges from 20 to a maximum of 80, and its clinical interpretation of anxiety is very low (20–35), low (36–45), average (46–55), high (56–65), or very high (65–80) [17].

A medical height gauge and a mechanical doctor's scale were used to measure height and weight for calculating the Body Mass Index (BMI) before and after the intervention.

An adapted version of the Astrand-Rhyming Sub-Maximal Effort Test [18,19] was used to estimate the maximal oxygen consumption (VO2 max) using a bicycle ergometer. This method is based on the relationship between heart rate recovery (HRR) and oxygen consumption reserve. In our study, we measured heart rate with a POLAR FT2® heart-rate monitor every minute (starting at rest). The work intensity began at 15 watts with 60 rotations per minute (RPM) and increased by 15 watts every minute to reach a target of 55% of the theoretical maximum heart rate for the age (220 bpm less age in years). When the adolescent reached this target, he/she continued to cycle for an additional five minutes. We averaged the heart rate from the two last minutes of the test.

### 2.5 Correlation between attendance rate and reduction of depression in exercise group

We looked at the correlation between the attendance rate and the difference in the reduction of depression in the exercise group. There was no correlation (for HADS D, r = −0.09, *p*-value = 0.71, and for SDS, *r* = 0.33, p-value = 0.15; r stands for Pearson cross-product correlation coefficient).

### 2.6 Sample size

As reported in the co-submission study [1], to measure variance in depression scores in HADS, the required sample size should be at least 18 participants per group in order to detect a difference of 2.4 at the 0.05 significance level with a power of 82%, and assuming an effect size of 1.0. In our data after interventions, the observed difference was 3.1 between the two groups of 20 participants; the size of our study has a power of 87% to detect such a treatment effect.

## Ethics Statement

Ethics authorization was received from the Ethics Committee of the Faculty of Medicine of the Université catholique de Louvain, and from the local Ethics Committee of Epsylon ASBL (N°. CEHF-2016/17FEV/060-B403201627586). Written informed consent according to the Declaration of Helsinki and medical history were required from the legal guardians of people aged less than 18 years of age, in addition to written consent from each participant.

## CRediT Author Statement

**Arnaud Philippot:** contributed to the study design, was involved in the two arms of the trial at all steps, and wrote the first draft of the manuscript; **Vincent Dubois:** was responsible for all medical procedures and logistical organization of the project in the Area+ hospital; **Alexandre Beine:** supervised the diagnostic interviews and psychiatric care; **Kate Lambrechts** and **Denis Grogna:** collected anamnestic data and contributed to the process of concealing the identities of participants; **Kate Lambrechts, Denis Grogna, Ugo Jonckheer** and **Wagdan Chakib:** were involved in the two arms of the trial; **Yannick Bleyenheuf:** contributed to the study design, manuscript draft and advised RCT procedure; **Annie Robert:** made the statistical analysis and contributed to the manuscript draft; **Anne G De Volde:** made the study design, verified the exercise and control arms, made all scoring blindly, provided results on an anonymous basis, and wrote the manuscript. At the final step, all authors had access to the study data that support the publication.

## Declaration of Competing Interest

AG De Volder is Senior Research Associate at the Belgian National Fund for Scientific Research. This study was supported by sponsorship of the Baillet-Latour asbl Funds (Belgium) to Prof. V. Dubois in the AREA+ hospital. The authors declare that they have no known competing financial interests or personal relationships which have or could be perceived to have influenced the work reported in this article.

## Data Availability

Dataset for: Exercise effects in psychiatry for adolescents on depression, anxiety and physical outcome (Original data) (PsychArchives).
